# Impact of Implementing Female Genomic Selection and the Use of Sex-Selected Semen Technology on Genetic Gain in a Dairy Herd in New Zealand

**DOI:** 10.3390/ijms26030990

**Published:** 2025-01-24

**Authors:** Craig Mckimmie, Mehrnush Forutan, Håvard Melbo Tajet, Alireza Ehsani, Jonathan Hickford, Hamed Amirpour

**Affiliations:** 1 Samen NZ Ltd., Morrinsville 3300, New Zealand; craig@samen.co.nz; 2 Centre for Animal Science, Queensland Alliance for Agriculture and Food Innovation, The University of Queensland, Brisbane, QLD 4072, Australia; m.forutan@uq.edu.au; 3 Geno SA, Storhamargata 44, 2317 Hamar, Norway; havard.melbo.tajet@geno.no; 4 Roslin Institute Building, Easter Bush, Scotland Rural College, Midlothian EH25 9RG, UK; alireza.ehsani@sruc.ac.uk; 5 Faculty of Agricultural and Life Science, Lincoln University, Lincoln 7647, New Zealand; jon.hickford@lincoln.ac.nz

**Keywords:** animal breeding, dairy cattle, genomic testing, genetic gain, New Zealand, sexed semen

## Abstract

Genomic selection (GS) has changed cattle breeding, but its use so far has been in selecting superior bulls for breeding. However, its farm-level impact, particularly on female selection, remains less explored. This study aimed to investigate the impact of implementing GS to identify superior cows and using artificial mating of those cows with sex-selected semen in a New Zealand Holstein-Friesian (HF) dairy herd (*n* = 1800 cows). Heifers (*n* = 2061) born over four consecutive years between 2021 and 2024 were genotyped and their genomic breeding values (GBVs) were estimated. These heifers were ranked based on the Balanced Performance Index (BPI; DataGene, Dairy Australia) Lower-performing cows producing less than 15 L/day (or 20 L/day for older cows) and those with severe mastitis were culled. Cows were mated with HF genetics based on production and udder breeding values, while lower-performing cows were mated to beef genetics. Milking adult cows were mated to bulls with similar BPI value. Annual genetic change was measured using Australian breeding values (ABVg) for milk fat production (FAT), protein production (PROT), fertility (FER), Mastitis Resistance (MAS), and BPI. The genetic merits of the heifers improved annually, with BPI increasing from 136 to 184 between 2021 and 2023, corresponding to a financial gain of NZD 17.53 per animal per year. The predicted BPI gain from 2023 to 2026 is expected to rise from 184 to 384, resulting in a financial gain of NZD 72.96 per animal per year. Using sex-selected semen on the top 50% of BPI-rated heifers in 2024 further accelerated genetic gain. Predicted BPI values for progeny born in 2025 and 2026 are 320 and 384, respectively. These findings revealed that the female GS, combined with sex-selected semen from genomically selected bulls, significantly accelerates genetic gain by improving the intensity and accuracy of selection. The approach achieves genetic progress equivalent to what traditionally would have required eight years of breeding without female GS, and has potential to improve dairy herd performance and profitability.

## 1. Introduction

The use of genomic selection (GS) has enabled genetic gain, with substantial improvements in productivity and fertility traits in dairy cattle [1]. The inherent flexibility of GS enables shorter generation intervals and higher selection intensities, which are key components for enhancing breeding efficiency [2]. While genetic progress through genomic testing and the selection of young bulls has markedly improved the performance of dairy cattle compared to traditional progeny testing approaches [3], female calves can have an even greater impact on farm viability and production when they enter the herd and contribute to improved milking performance. The use of sexed semen in combination with genomic testing of females has been highlighted as a strategy to increase the accuracy of predicted breeding values and rate of genetic change and can be used in synergy with other management tools and strategies [4].

Selecting cows with high genomic estimated breeding values (GBVs) is economically advantageous, highlighting the importance of cost–benefit strategies in breeding decisions [3,5]. Moreover, reproductive technologies such as the use of sex-selected semen for artificial insemination offer the potential to enhance the integration between dairy and beef production, as well as increase farm profitability effectively. With this technology, low genetic merit cows for dairy production can be mated to beef genetics, while superior dairy genetics can be mated to female sex-selected elite dairy genetics [6]. The combined use of the GS of females and the use of reproductive technologies has increased annual monetary genetic gain in dairy breeding programs [7].

Research has also revealed that female genomic testing can improve the accuracy of genomic breeding values in dairy cattle [8]. Farmers are increasingly applying genomic testing to females, rather than just male breeding stock. However, this gain in accuracy can be low if the training populations used for designing GS systems are small [8]. Furthermore, diverse and potentially unquantifiable environmental effects on production performance may also limit the consistency of genomic predictions. Taken together, it has been estimated that the combination of GS and the use of sex-selected semen can lead to a threefold increase in genetic gain and a 73% increase in the accuracy of male selection [9].

Economic evaluations emphasize the importance of integrating genomic testing with reproductive strategies to optimize economic returns and reduce genetic lag, which is the delay in genetic improvement within a herd due to the use of older or less genetically advanced animals [10]. From an economic perspective, it has however been confirmed that combining GS with sex-selected semen is cost-effective [11,12]. The approach requires GS strategies and reproductive technologies to be managed to achieve the best outcomes in terms of genetic and economic gains [13], with one report suggesting that the widespread adoption of sex-selected semen technology should be limited to well-managed herds that already achieve acceptable fertility performance, so as to maximize economic benefit [14]. However, genetic progress in dairy farming is crucial if one is to realize improved productivity and profitability. Genomic analysis is becoming a key tool in enabling this progress, as it allows for the selection of superior breeding stock, while mitigating the negative effects of inbreeding depression [15]. This progress has been shown to increase profits, with improved management further enhancing its impact [16].

Evaluating genetic improvement at the farm level assists farmers in developing improved mating strategies. In this respect, the integration of sex-selected semen into artificial insemination programs has been reported to have positive economic effects, as it increases the proportion of female offspring, which can lead to improved herd genetics and potentially higher profitability [17,18]. In this case study, we aimed to evaluate the genetic and economic benefits of using sex-selected semen in conjunction with GS, in a previously un-recorded and poorer-performing HF dairy herd in New Zealand.

## 2. Results

The average genetic gain for the four key traits along with BPI and ASI (Australian Selection Index) for all genotyped animals born from 2021 to 2024 are reported in Table 1. Notably, an increase in genetic gain has been achieved for animals born in 2024 across all four key traits, when compared to the outcome before integrating GS and sex-selected semen use, where the only gain was observed for FAT. The effect of GS was observed in the increased genetic gain (step change) achieved for 2024 and predicted for 2025 compared to the genetic gain achieved 3 years prior to GS. Although not ideal, the study’s design constraints needed the approach of comparing genetic gain between years within a single herd. This method provides a preliminary assessment of genetic change over time. However, more sophisticated statistical methods and multi-herd comparisons would yield more robust insights into the impacts of GS and sex-selected semen. Only one animal with a BPI > 150 has been culled for mastitis in four quarters; she was the lowest mastitis resistance genomic BV heifer in the line. No animals with BPI > 150 were culled on production, as would be expected.

Out of the 818 females selected for mating in 2025 with sex-selected semen (High group), 147 were born in 2021 (representing 17.9% of the cows mated to sexed semen), 259 were born in 2022 (representing 31.6% of the cows mated to sexed semen), and 409 were born in 2023 (representing 50.0% of the cows mated to sexed semen). This indicates an increasing trend for younger cohorts to be represented in the selection process. The average genetic gain per year for the first three years was 16 and 10 points in BPI and ASI, respectively. The improvement observed in the matings born in 2024, following the integration of GS and the use of sex-selected semen, was projected to yield a BPI and ASI of 312 and 142, an increase of 128 and 48 points in BPI and ASI, respectively. Such improvements equated to eight years of genetic gain for BPI and 5.3 years for ASI without using sex-selected semen and GS, surpassing the annual genetic gain observed prior to the implementation of female GS.

Table 2 reveals an increase in all traits for the group selected for breeding (High group) compared to those mated to beef genetics (Low group). The results reveal a steady annual genetic gain of 17 (229-177) and 14.3 (138-95) points in BPI when cows were mated with sex-selected semen and beef straws, respectively (Table 2).

Table 3 summarizes the population genetic metrics observed in the genomically selected cattle. The metrics include mean allele frequency, mean and median PI_HAT (a measure of genetic relatedness), observed and expected heterozygosity, and the inbreeding coefficient (FIS).

The moderate level of relatedness between the cows (mean and median PI_HAT values of 0.1855 and 0.1773, respectively), and the moderate observed heterozygosity (0.2654), coupled with a negative FIS value, suggest a rich genetic diversity within the herd. This is beneficial for disease resilience and productivity optimization (Table 3). Although deviations from HWE were identified at some loci, they were not excluded, as the analysis aimed to assess overall genetic diversity rather than locus-specific variation. This level of heterozygosity is important for the herd’s health and long-term sustainability and indicates that the current genetic management strategies have been effective at preventing inbreeding and supporting genetic diversity.

**Figure 1:**
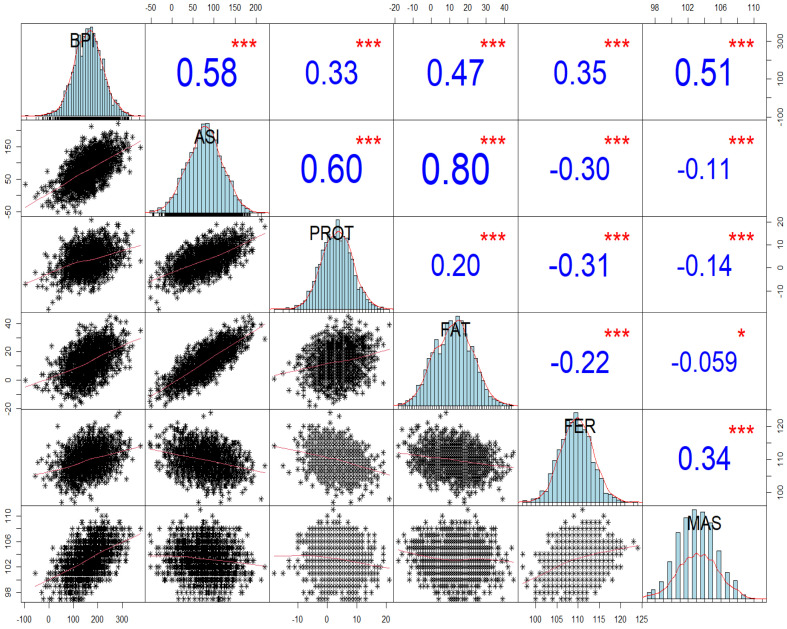
Above the diagonal line are reported the Pearson correlation coefficients (r values) between BPI (Balanced Performance Index; DataGene, Dairy Australia), ASI (Australian Selection Index), and Australian Breeding Values (ABVs) for different traits in genotyped heifers. Below the diagonal line are the scatterplots depicting the relationships between the traits. Correlation values are shown in blue, indicating the intensity of the correlations (weak, moderate, or strong). The *p*-values are in red, but they only represent the significance value and not the intensity of the correlations. Significance levels are indicated as follows: * *p* < 0.05, *** *p* < 0.001.

Figure 1 depicts the correlations between BPI, ASI, and four key traits: FAT, PROT, MAS, and FER in genotyped heifers. These results reveal the vital role of the selection index (BPI) versus the production index (ASI) on the important traits of FER and MAS. There is a balance of correlation between BPI with the production traits, MAS, and FER, ranging from 0.33 to 0.58.

### The Economic Advantage of the Use of GS of Cows and Sex-Selected Semen

The economic benefits of incorporating GS and sex-selected semen are substantial. The achieved BPI for animals mated with the female genomic testing strategy born in 2024 is 312, resulting in an economic gain of approximately NZD 341.84 per animal. This economic gain is attributed to the enhanced genetic merit achieved through GS and use of sex-selected semen, with these increasing both selection intensity and accuracy, leading to faster genetic progress. The substantial genetic gains projected for 2026, equivalent to 12.5 years of genetic progress for BPI and 7.8 years for ASI, highlight the economic value of these technologies in improving the performance and profitability of dairy herds.

## 3. Discussion

### 3.1. Economic Weight of Each Trait in the Balanced Performance Index (BPI)

The Balanced Performance Index (BPI) is an economic index that integrates various traits to drive improvements in the lifetime contribution of dairy cows to a farm business. For HF cattle, the economic weighting for protein yield is 6.76, fat yield has an economic weight of 2.08, and milk yield is −0.082. The negative economic weighting for milk yield reflects the associated costs of producing higher milk volumes, such as increased feed requirements and potential health risks, without corresponding economic returns. Other key traits include survival (7.2), daughter fertility (6.94), somatic cell count (0.69), mastitis resistance (6.75), milking speed (5.02), temperament (3.60), mammary system (2.76), udder depth (0.82), overall type (1.32), pin set (0.78), and feed saved (0.1927) (see https://www.datagene.com.au/genetics/indices-and-traits/ (accessed on 10 July 2024) for further details). These weightings reflect a balanced approach to valuing production, health, and type traits in the BPI breeding objectives.

### 3.2. Implications of Genetic Diversity for Herd Productivity and Sustainability

The moderate observed heterozygosity and the negative FIS value indicate that the current genetic management strategies are effective at minimizing inbreeding and maintaining a diverse genetic base. This diversity is beneficial for disease resilience, as heterozygosity has been associated with improved immune function and resistance to diseases such as mastitis and bovine respiratory disease [19]. Increased genetic variation reduces the likelihood of disease outbreaks and promotes adaptability to environmental stressors, which are critical for herd sustainability [20].

Additionally, herds with lower inbreeding coefficients have been shown to exhibit better fertility, milk production, and overall health [21]. The slight excess of heterozygosity observed in this study suggests that GS strategies can successfully balance genetic gain with maintaining genetic diversity [19]. This balance is essential to mitigating inbreeding depression, which can adversely affect economically important traits such as fertility and milk components [22]. By continuing to optimize sire selection, culling less fertile or lower-performing animals, and employing genomic tools to monitor genetic relationships, this herd can sustain long-term productivity while minimizing the risks associated with reduced genetic diversity.

In subsequent generations, sire selection and the fact that lower-fertility animals often fail to get in calf and are culled more rapidly, would lessen the need for GS for this trait. This approach uses the BPI that optimizes fertility across the herd and could increase both productivity and economic sustainability. By maintaining genetic diversity and employing GS strategies, the herd can achieve long-term health and productivity goals while minimizing risks such as inbreeding and fertility challenges.

### 3.3. Accelerated Genetic Gains Through GS

The BPI values had balanced correlations with key traits such as FAT, PROT, MAS and FER. This suggests that BPI is an effective predictor for these traits in HF heifers. There was, as expected, positive correlation between BPI and all four traits (FAT, PROT, MAS, and FER), while FAT and PROT exhibited negative correlations with MAS and FER.

This study confirmed the efficiency of GS in accelerating genetic gains in this herd. The increase in BPI and ASI post-GS resembled findings from earlier studies in dairy cattle [9,23,24,25]. The results of this study revealed a substantial increase in the rate of genetic gain, namely a 128-point increase in BPI post-GS, which aligned with the findings of McHugh et al. [9], who also noted that GS could potentially greatly increase the rate of genetic gain. Further support comes from a study of Montbéliarde cows in France, where the use of genomic information over pedigree data significantly improved mate allocation, leading to enhanced genetic merit in progeny [26]. This parallel in findings with the HF heifers studied here suggests global applicability and effectiveness for GS in dairy cattle breeding programs

The largest amount of genetic gain observed after GS and sex-selected semen use came from yearling matings. Traditionally, in New Zealand, HF yearlings mated to Jersey bulls do not have progeny entering the herd. Our study focused on GS in a New Zealand dairy herd, highlighting the benefit of using sex-selected semen in dairy beef production. This aligns with the New Zealand dairy industry’s goals of increasing production efficiency and genetic gain. The use of sex-selected semen minimizes the production of typically unwanted male dairy-breed calves and reduces the risks associated with dystocia, directly affecting herd health and productivity. Using sex-selected semen has been estimated to increase the rate of genetic gain by 15% [4], and this aligns with our observations in HF heifers.

### 3.4. Economic and Genetic Impacts of Sexed Semen Use

The economic benefits of using sex-selected semen seem to be profound. Previous findings also indicate that the benefit of using sex-selected semen in dairy herds is influenced by the baseline fertility of the herd [27]. In high-fertility scenarios, the use of sex-selected semen in both heifers and cows revealed a favorable economic advantage, which aligns with our projections of increased revenue gains from genomic testing in under-performing New Zealand dairy herds. For instance, annual revenue gains attributable to sex-selected semen and genomic testing are projected to significantly enhance the profitability of dairy herds. Studies, such as De Vries et al. [28], provide critical quantification of the economic gains, indicating that the rate of genetic gain could be increased by up to 15%.

Genetically, the use of sexed semen supports accelerated genetic gain by selectively increasing the proportion of replacement heifers while reducing the risks of dystocia associated with calving. However, challenges such as the higher initial costs and variable conception rates emphasize the need for robust herd management strategies to maximize its effectiveness.

Future research should incorporate comprehensive economic analyses that directly quantify the trade-offs associated with sexed semen use. This includes modeling potential impacts across varying herd fertility levels and management systems to provide actionable insights for the dairy industry.

### 3.5. Economic Ramifications

In this herd, approximately four hundred heifers were selected annually as milking herd replacements, representing an estimated annual replacement rate of 25% of the total milking herd. The genetic gain obtained by implementing GS was 128 BPI, equivalent to approximately 140.8 New Zealand Dollars (NZD). Consequently, the annual revenue gains attributable to genomic testing for this herd amounts to AUD 51,200 (approximately NZD 56,320) per year for each age group. Over three years, the genetic gain could total AUD 153,600 (approximately NZD 168,960). The exchange rate used for these calculations was 1 AUD = 1.1 NZD, as reported by Google on 17 October 2024. If this benefit could be replicated across the whole New Zealand dairy herd (4,701,596 cows in 2024) the economic benefit could be NZD 2,647,938,868 or approximately NZD 2.65 billion added to New Zealand economy. This compares to the current rate of genetic gain in New Zealand of NZD 19.04 per cow per year for HF cattle [29], which gives a potential improvement of NZD 89,518,388 for all cows in New Zealand, and over four years this would amount to a gain of NZD 358,073,552.

Other research [9,20,21] has laid a foundation illustrating how GS can increase genetic gain in dairy breeding programs. These studies have highlighted the importance of expanding the reference population through more extensive genotyping to improve genetic gain and reduce inbreeding rates [21]. Such advancements, including the development of lifetime genetic–economic indices, point toward a future where GS plays a vital role in improving dairy production. These findings not only contribute to the ongoing efforts to refine dairy cattle breeding strategies through GS, but also emphasize the need for comprehensive approaches that consider both milk production efficiencies and the health traits of dairy cattle. However, a previous study [30], reported that GS could result in a marked increase in inbreeding. Accordingly, genetic diversity should be considered when implementing GS approaches. To mitigate the risks of increased inbreeding associated with GS, several strategies can be employed. Expanding the reference population through extensive genotyping can help maintain genetic diversity and reduce inbreeding rates. Implementing optimal mating plans that consider genetic relationships and aim to minimize inbreeding while maximizing genetic gain is crucial. Regularly monitoring inbreeding levels and adjusting breeding strategies accordingly to prevent excessive inbreeding is essential. Incorporating crossbreeding strategies can introduce new genetic material and enhance genetic diversity. Utilizing genomic tools to identify and manage inbreeding by selecting animals with lower inbreeding coefficients is also recommended.

By adopting these strategies, dairy farmers can leverage the benefits of genomic selection while mitigating the potential risks associated with increased inbreeding. This balanced approach ensures sustainable genetic improvement and long-term viability of dairy herds.

### 3.6. Limitations and Future Directions

One limitation of our study is the exclusion of the additional costs and risks associated with the use of sex-selected semen. However, previous research has modelled these economic impacts. For instance, De Vries et al. [28] explored the potential effects of sexed semen on the dairy industry’s structure, considering both costs and benefits. These include higher costs per straw and potential reductions in pregnancy rates per service. Future studies should incorporate these factors into their economic analyses to provide a more comprehensive evaluation of the cost-effectiveness of using sex-selected semen in genetic improvement programs. Additionally, the economic and genetic impacts of sexed semen use are discussed further in Section 3.5. Previous studies have quantified the potential annual revenue gains and other economic ramifications, providing valuable insights into the cost–benefit analysis of this technology [19,24].

## 4. Materials and Methods

### 4.1. The Dairy Herd

This herd was established in 2019 by purchasing of New Zealand Holstein-Friesian cows from low-input production systems. These cows did not transition well to increased feeding on a new property, with most having a poor response with high levels of mastitis, poor production, poor fertility, and udder collapse. In 2019, the cows were randomly mated to North American HF dairy sires to improve their suitability for the new production system, but the farmer saw mixed results, with some of the progeny showing improved response to feeding, and some showing no improvement. The farmer had previously only collected phenotypic data for milk yield and received data for milk solids per cow from their dairy company. Herd replacements that produced less than 15 L/day of milk were culled, as were older cows that produced less than 20 L/day. Any cows with severe mastitis were culled. Severity was assessed based on persistently high somatic cell counts (SCC) observed in milk samples. As there were minimal phenotypic data collected in this herd, the decision was made to make all future selection and breeding decisions based on an Australian genomics analysis due to their extensive heifer phenotyping undertaken by DataGene and Dairy Australia (https://www.datagene.com.au/data/ginfo/) (accessed on 10 July 2024).

A total of 2061 females born in 2021 (being milked, *n* = 484), 2022 (*n* = 523), 2023 (*n* = 617), and 2024 (*n* = 437) were used from the split calving herd in Canterbury, New Zealand. In this herd, calving is divided across autumn and spring to optimize production and pasture availability. They evaluated each heifer calf using genomic Australian breeding values (ABVg) from DataVat [31] for four key traits, including FAT, PROT, FER, MAS, and for BPI. The use of ABVg in this study, despite being conducted in New Zealand, was due to the lack of a GS index in New Zealand. ABVg was chosen for its robustness and alignment with the breeding objectives of this study. Additionally, high genetic correlations between Australian and New Zealand breeding values for key traits, including milk production, fertility, and health traits, validate the applicability of ABVg in this context [32].

Cow fertility was measured as the percentage of an animal’s daughters pregnant by six weeks after the mating start date, compared to the base cow [31], and the Balanced Performance Index (BPI) encompasses several core traits: fat (FAT), protein (PROT), and mastitis resistance (MAS), so as to evaluate an animal’s economic merit in a dairy production context. FAT estimates an animal’s ability to produce kilograms of fat in milk, and PROT ABV estimates its ability to produce kilograms of protein. The MAS ABV indicates an animal’s resistance to mastitis, with higher values representing healthier cows. The BPI synthesizes these production and health metrics alongside management traits, delivering a robust index that describes the animal’s potential for economic return and genetic advancement within a modern dairy operation. For an animal to get an ABVg, it needed to have a recorded genotype, a unique national ID, a valid birth date, a breed code, and a known sire that had also been given an ABV.

The 2061 HF heifers, upon evaluation, were divided into two breeding groups: the top 50% (High, *n* = 1250), mated with sex-selected semen for heifer replacements, and a group with the bottom 50% BPI (Low, *n* = 825), mated with Wagyu beef straws.

### 4.2. Cow Genotyping

Tissue Sampling Units (TSUs) were used for collecting samples from the 1338 heifers, adhering to strict ethical guidelines to ensure animal welfare (Animal Welfare Act 1999, New Zealand Government). The TSU device collects a small ear notch sample with a single squeeze of the applicator, ensuring minimal stress to the animal. The tissue sample is sealed in a bar-coded vial, preserving high-quality DNA for genomic testing while minimizing the risk of contamination. Samples were stored at room temperature and processed promptly to maintain DNA integrity. The 2061 cows were genotyped using ThermoFisher Scientific’s Axiom Bovine Genotyping Array (BovMDv3 array) by Xytovet (Xytivet Pty Ltd., Perth, Western Australia, Australia).

### 4.3. Population Structure and Statistical Analyses

Population genetic statistics, including K-means clustering, pairwise relatedness, Hardy–Weinberg Equilibrium (HWE), mean allele frequency, genomic heterozygosity, and inbreeding coefficient (FIS), were computed using PLINK version 1.9 [33]. Genomic heterozygosity was estimated by calculating the proportion of heterozygous loci for each individual. The inbreeding coefficient (F) was determined using the following formula:

$$F=1-\frac{O}{E}$$

where O is the observed number of homozygous loci, and E is the expected number of homozygous loci under Hardy–Weinberg equilibrium.

To evaluate the relationships between production traits and selection indices, Pearson correlation coefficients were calculated using RStudio version 4.4.2 [34]. These correlations assessed the strength and direction of the relationships between the BPI, ASI, and key traits such as FAT, PROT, FER, and MAS. To assess the impact of the combined approach (GS and sex-selected semen) on the overall genetic improvement of the herd, we performed Analysis of Variance (ANOVA) tests to compare the differences in ABVg (for PROT, FAT, FER, and MAS), BPI, and the Australian Selection Index (ASI) between two groups. The Tukey Honest Significant Difference (HSD) post-hoc test was employed to investigate pairwise differences between the two groups using RStudio [34].

### 4.4. Statistical Analysis to Estimate Genomic Breeding Values

The genomic breeding values (GBVs) were calculated and estimated using statistical methods as detailed in previous studies [35] by DataGene. The approach involves the application of best linear unbiased prediction (BLUP) models integrated with genomic information. Specifically, a single-step genomic BLUP (ssGBLUP) method was used to combine pedigree, phenotypic, and genomic data into a unified analysis. This method allows for the estimation of breeding values by leveraging the marker information provided by genotyping.

DataGene’s procedures follow those outlined by Nieuwhof et al. [35], which include the use of an SNPBLUP model for direct genetic values (DGVs) and blending to combine various sources of data. The process involves genotype quality assurance, imputation of missing genotypes, estimation of DGVs using SNPBLUP, and blending with pedigree and phenotypic information to derive the final GBVs.

The SNPBLUP model estimates the DGV for an individual i using the following equation:

$$g_{i}=\sum_{K=1}^{\rho }\chi_{ik}\beta_{k}$$

where $g_{i}\;$ is the direct genetic value for individual i; $\chi_{ik}\;$ is the genotype of individual i at SNP k; $\beta_{k}\;$ is the effect of SNP k; and ρ is the total number of SNPs.

## 5. Conclusions

This study demonstrates that implementing female GS and the strategic use of sexed semen can create a step-change increase in genetic merit in a dairy herd. The increase in BPI from 2023 to 2024 was 128 (69.6%) and ASI was 48 (51.1%), and post-genomic testing highlights the effectiveness of these breeding strategies. While genomic selection and sexed semen are likely to boost productivity and profitability, it is important to note that these effects may vary across herds with differing baseline conditions, such as fertility, milk production, herd size, genetic variability, and management practices. These advancements could boost productivity and profitability by increasing production, reducing mastitis, and improving fertility outcomes (such as increased daughter fertility), and thus would also have a vital role in addressing concerns about animal welfare and sustainability.

## Figures and Tables

**Table 1:** A comparison of the BPI, ASI, and ABVs for all the cows born across years before and after the implementation of GS and sexed semen in the herd.

Year Born	BPI ^1^	ASI ^2^	Fat (kg) ^3^	Prot (kg) ^4^	Fertility ^5^	MAS ^6^
2021	136	65	9.2	3.6	109.3	102.3
2022	161	73	11.8	3.1	110.3	103.7
2023	184	94	17.3	3.8	110.1	103.5
2024	312	142	19.3	15.6	112.1	105.1
2025 (Predicted)	320	135	28.7	8.6	112.5	105.7
2026 (Predicted)	384	172	36.1	13.4	111.2	106.2

^1^ BPI (Balanced Performance Index): An economic index that blends production, health, and type traits to assess overall profitability potential. ^2^ ASI (Australian Selection Index): A production-based index that ranks animals on fat, protein, and milk yield profitability. ^3^ Fat (kg): Fat yield in kilograms, estimating the heifer’s genetic potential to produce fat in milk. ^4^ Prot (kg): Protein yield in kilograms, estimating the heifer’s genetic potential to produce protein in milk. ^5^ Fertility (Fertility ABV): An estimate reflecting the likelihood of a heifer’s daughters becoming pregnant within six weeks of the mating start date. ^6^ MAS (Mastitis Resistance ABV): Mastitis resistance score, with higher values indicating stronger resistance to mastitis.

**Table 2:** Genetic change in key mated heifer traits before and after implementation of a genome selection-based breeding approach.

Birth Year	Selection Group	BPI ^1^	ASI ^2^	Prot (kg) ^3^	Fat (kg) ^4^	Fertility ^5^	MAS ^6^
2021	High group (Sexed Semen;n=246)	177	81	4.6	11.2	110.6	103.2
	Low group (n=243)	95	49	2.5	7.1	108.1	101.5
2022	High group (Sexed Semen; n=262)	206	90	3.7	14.5	110.8	104.6
	Low group (n=261)	116	56	2.2	7.1	108.8	102.6
2023	High group (Sexed Semen; n=312)	229	113	5.7	20.6	110.2	104.4
	Low group (n=313)	138	76	1.8	14.1	108.9	102.7

^1^ BPI (Balanced Performance Index): An economic index that blends production, health, and type traits to assess overall profitability potential. ^2^ ASI (Australian Selection Index): A production-based index that ranks animals on fat, protein, and milk yield profitability. ^3^ Prot (kg): Protein yield in kilograms, estimating the heifer’s genetic potential to produce protein in milk. ^4^ Fat (kg): Fat yield in kilograms, estimating the heifer’s genetic potential to produce fat in milk. ^5^ Fertility (Fertility ABV): An estimate reflecting the likelihood of a heifer’s daughters becoming pregnant within six weeks of the mating start date. ^6^ MAS (Mastitis Resistance ABV): Mastitis resistance score, with higher values indicating stronger resistance to mastitis.

**Table 3:** Summary of population genetic metrics for the genomically selected cattle.

Metric	Value
Mean Allele Frequency ^1^	0.1938
Mean PI_HAT (IBD) ^2^	0.1855
Median PI_HAT (IBD) ^2^	0.1773
Range PI_HAT (IBD) ^2^	0–0.9027
Mean Observed Heterozygosity (HWE) ^3^	0.2654
Mean Expected Heterozygosity ^4^	0.2569
FIS ^5^	−0.0330

^1^ Mean Allele Frequency: The average frequency of alleles observed across all loci. ^2^ PI_HAT (IBD): Proportion of Identity by Descent (IBD), with PI_HAT values indicating the degree of genetic relatedness; higher values suggest closer relationships. Mean PI_HAT (IBD): The average PI_HAT across all pairwise comparisons. Median PI_HAT (IBD): The median PI_HAT across all pairwise comparisons. Range PI_HAT (IBD): The minimum and maximum PI_HAT values observed. ^3^ Observed Heterozygosity (HWE): The proportion of heterozygous individuals observed in the population, calculated under Hardy–Weinberg Equilibrium (HWE). ^4^ Expected Heterozygosity: The expected proportion of heterozygous individuals under Hardy–Weinberg Equilibrium. ^5^ FIS (Inbreeding Coefficient): An estimate of inbreeding within individuals relative to the total population; a negative value (e.g., −0.0330) indicates a slight excess of heterozygosity.

## Data Availability

The original data used in this paper are available upon request by contacting the corresponding author.
